# Evaluation of the effects of formulation, food, or a proton-pump inhibitor on the pharmacokinetics of glasdegib (PF-04449913) in healthy volunteers: a randomized phase I study

**DOI:** 10.1007/s00280-018-3748-8

**Published:** 2018-12-10

**Authors:** Naveed Shaik, Brian Hee, Hua Wei, Robert R. LaBadie

**Affiliations:** 1 Pfizer Inc., Pfizer Oncology, Pfizer Global Product Development, 1055 Science Center Drive, San Diego, CA 92121 USA; 2 Pfizer Inc, Shanghai, China; 3 Pfizer Inc, Groton, CT USA

**Keywords:** PF-04449913, Glasdegib, Pharmacokinetics, Food effect, Formulation, Proton-pump inhibitor

## Abstract

**Purpose:**

To demonstrate the bioequivalence of the planned maleate salt-based commercial glasdegib tablet formulation [International Council for Harmonization (ICH) glasdegib] to the clinical di-hydrochloride (di-HCl) salt-based glasdegib formulation (di-HCl glasdegib). Additionally, to estimate the effects of a high-fat, high-calorie meal and proton-pump inhibitor (PPI) on the pharmacokinetics of ICH glasdegib.

**Methods:**

This Phase I open-label study (ClinicalTrials.gov: NCT03130556) enrolled 24 healthy subjects to receive two different tablet formulations of single-dose 100-mg glasdegib under fasted conditions. A subset of healthy volunteers (*n* = 12) received single-dose 100-mg ICH glasdegib following a high-fat, high-calorie meal or concurrently with a PPI (rabeprazole).

**Results:**

The adjusted geometric mean ratio (ICH glasdegib:di-HCl glasdegib) and 90% confidence intervals (CI) of area under the plasma concentration–time curve from time zero to infinity (AUC_inf_) and maximum plasma concentration (*C*_max_) were 104.0% (99.7‒108.5%) and 101.6% (96.1‒107.4%), respectively, within the acceptance range for bioequivalence (80‒125%). The adjusted geometric mean ratio (90% CIs) for AUC_inf_ and *C*_max_ under fed conditions were 84.3% (78.6‒90.6%) and 69.0% (61.8‒77.0%), respectively, relative to fasted conditions. When ICH glasdegib was administered concurrently with the PPI, the adjusted geometric mean ratio (90% CI) of AUC_inf_ and *C*_max_ were 100.6% (93.2‒108.6%) and 80.5% (70.7‒91.6%), respectively, relative to fasted conditions. Glasdegib was generally well tolerated under all conditions studied.

**Conclusions:**

The ICH glasdegib tablet formulation was bioequivalent to the clinical di-HCl formulation under fasted conditions. A high-fat, high-calorie meal or concurrent PPI treatment had a minimal effect on glasdegib exposure, and was not considered clinically meaningful.

## Introduction

Glasdegib (PF-04449913), an oral, small-molecule inhibitor of the smoothened receptor, selectively inhibits the Hedgehog signaling pathway [1]. Aberrant Hedgehog pathway signaling is associated with a variety of hematologic malignancies, and activation of the pathway is implicated in tumor formation, cancer progression, and drug resistance [2]. Preclinical studies have demonstrated glasdegib directly inhibits the growth of leukemic cells and is synergistic with chemotherapeutic agents [3, 4]. Glasdegib is currently in Phase III clinical development (ClinicalTrials.gov, NCT03416179) for the treatment of acute myeloid leukemia (AML); it is also being evaluated (ClinicalTrials.gov, NCT02367456) for high-risk myelodysplastic syndrome (MDS). Based on prior Phase I, single-agent, dose-escalation studies in patients with cancer, the recommended dose for glasdegib in patients with AML or MDS is 100 mg once daily (QD) [5].

Glasdegib is formulated as a 100-mg immediate-release oral tablet. Phase I/II clinical trials utilized a di-hydrochloride monohydrate (di-HCl glasdegib) formulation tablet, developed to determine the efficacy and safety of glasdegib in these early trials [6, 7]. However, the di-HCl glasdegib formulation was not considered optimal for further clinical development. During the drug development process, salt screening and optimization of the formulation are required to create a commercially viable tablet formulation. A previous study evaluated the use of physically stable, maleate salt-based glasdegib tablet formulations [8]. The study tested two novel maleate formulations with differing salt particle size, identifying them as bioequivalent to the di-HCl tablet formulation [8]. Additionally, the results of the study identified an acceptable range for active pharmaceutical ingredient particle size for future manufacture of maleate glasdegib tablet formulations. The proposed commercial maleate glasdegib tablet evaluated in the current report had changes to the drug load and changes in the percentage of some excipients, along with coloring and debossing.

Since glasdegib is orally administered, it is important to determine the pharmacokinetics (PK) in relation to meal consumption and concurrent medications, such as proton-pump inhibitors (PPIs), that might impact absorption. In vitro assessment determined the lipophilicity of glasdegib to be low (*c*Log*P* = 2.28), with glasdegib considered to be a Biopharmaceutical Classification System class II or IV drug (unpublished) [1]. The effect of a high-fat, high-calorie meal on the PK of the di-HCl formulation tablet and earlier maleate-salt formulations was previously investigated [8, 9]. Based on these studies, the recommendation was that glasdegib may be taken independent of food intake. The solubility of glasdegib, a weakly basic drug, is hydrogen (pH)-dependent, with decreasing solubility as pH increases. Therefore, glasdegib PK could theoretically be altered when concomitantly administered with drugs that elevate gastric pH, such as PPIs. Cancer patients frequently receive PPIs for the treatment or prophylaxis of concomitant conditions; understanding the potential effects of these drugs on the bioavailability of glasdegib is therefore important [10]. The effects of PPIs on the PK of the early maleate-salt glasdegib tablet formulations were previously evaluated [8]. Glasdegib dosing in the presence of PPIs resulted in a 12% increase in area under the plasma concentration–time curve (AUC) from time zero to infinity (AUC_inf_) and a 13% decrease in maximum plasma concentration (*C*_max_), indicating no clinically relevant effect of PPIs on glasdegib following a single 100-mg oral dose. Therefore, the current study aimed to estimate the impact of food and a PPI on the planned commercial formulation of glasdegib.

The present study evaluated a maleate salt formulation of glasdegib that is compliant with the International Council for Harmonization (ICH) of Technical Requirements for Registration of Pharmaceuticals for Human Use (ICH glasdegib) [11]. This formulation is intended to be the final commercial formulation. The primary objective of this study was to establish bioequivalence of the new ICH glasdegib tablet formulation to the di-HCl glasdegib formulation. The secondary aims were to determine the effects of a high-calorie, high-fat meal or a PPI (rabeprazole) on the plasma PK of single-dose ICH glasdegib. Additionally, the safety and tolerability of a single dose of glasdegib administered in fasted or fed states or in the presence of a PPI were evaluated. This study was not designed, nor did it aim to demonstrate BE or test for BE between fasted di-HCL and fed ICH maleate glasdegib.

## Materials and methods

### Study design

**Fig. 1:**
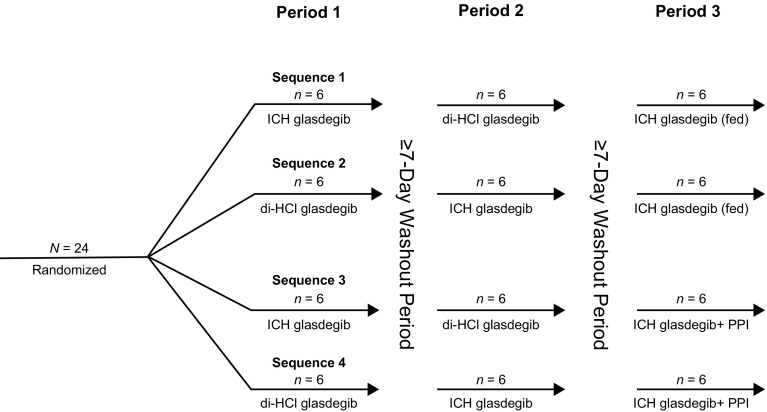
Treatment sequence. Healthy volunteers received 100 mg of glasdegib as a single, oral, instant-release formulation tablet. All doses were given in a fasted state (> 10 h) unless otherwise stated. The duration of each period was 6 days following glasdegib administration. *N* number of subjects, *PPI* proton-pump inhibitor (rabeprazole)

This was a Phase I, randomized, open-label, three-period, four-sequence, four-treatment study in healthy volunteers. Subjects were randomized to one of four treatment sequences (Fig. 1). All subjects received three treatments with a washout period of at least 7 days between each administration period. The duration of each administration period was 6 days with the exception of PPI treatment, which was 12 days (including 6-day follow-up after glasdegib administration). Glasdegib was administered with 240 mL of water, and water was withheld for 1 h both before and after administration.

The primary study objective was to determine the bioequivalence of the new ICH glasdegib formulation to the di-HCl glasdegib formulation (determined in periods 1 and 2) using a two-way crossover study design. Subjects received a 100-mg single dose of di-HCl glasdegib or ICH glasdegib, which was administered after an overnight fast of at least 10 h. No food was allowed for at least 4 h after glasdegib dosing.

The effects of food on the PK of ICH glasdegib were determined during period 3 in a subset of subjects (*n* = 12) using a one-way crossover study design. Following a 10-h fast, subjects consumed a recommended high-fat (~ 50% of total caloric content of the meal), high-calorie meal (~ 800 to 1000 calories with 150, 250, and 500–600 calories from protein, carbohydrate, and fat, respectively). Subjects received a 100-mg single dose of ICH glasdegib ~ 5 min after the meal was consumed. The reference for this evaluation was ICH glasdegib administered under fasted conditions during periods 1 and 2.

The effect of a PPI (rabeprazole) on the PK of ICH glasdegib was determined during period 3 in a subset of subjects (*n* = 12) using a one-way crossover study design. Following a 7-day washout period, rabeprazole 40 mg was administered daily for 7 days. On the day of PK sample collection (day 7 of rabeprazole administration), both rabeprazole and glasdegib were administered in the fasted state. Rabeprazole was administered 4 h before glasdegib. No food was allowed for 4 h following glasdegib administration. The reference for this objective was ICH glasdegib administered under fasted conditions (periods 1 and 2).

The primary PK endpoints were AUC_inf_ and *C*_max_ of glasdegib. Additional PK endpoints were AUC from time 0 to the last quantifiable concentration (AUC_last_), time for *C*_max_ (*T*_max_), plasma elimination half-life (*t*_½_), apparent oral clearance (CL/*F*), and apparent volume of distribution following oral dose (*V*_*z*_*/F*).

### Subjects

Eligible subjects were healthy females of non-childbearing potential and males 18‒55 years of age, with a body mass index (BMI) of 17.5‒30.5 kg/m^2^. Subjects were in good health based on medical history, physical examination (including blood pressure and pulse rate measurements), 12-lead electrocardiogram (ECG), and clinical laboratory tests. Subjects were excluded if they did not meet entry criteria, including if they had a known sensitivity to PPIs, donated blood within 60 days prior to dosing, had any condition possibly affecting drug absorption (e.g., gastrectomy, achlorhydria) or used prescription/non-prescription drugs and dietary supplements within 7 days or five half-lives [whichever was longer (with the exception of acetaminophen/paracetamol)], or had abnormal clinical laboratory tests for liver function. Subjects were also excluded if they had an ECG demonstrating the time from the beginning of the Q wave to the end of the T wave corresponding to electrical systole (QT) corrected for the heart rate interval > 450 ms or a time from ECG Q wave to the end of the S wave corresponding to ventricle depolarization (QRS) interval > 120 ms or had a family history of myocardial infarction, congenital long QT syndrome, torsades de pointes, or clinically significant ventricular arrhythmias.

### Ethical approval

The study protocol was approved by an independent institutional review board and was conducted in accordance with the Declaration of Helsinki and Good Clinical Practice guidelines. Informed consent was obtained from all individual participants included in the study. This trial is registered with ClinicalTrials.gov, identifier NCT03130556.

### Pharmacokinetic assessments and analysis

Blood samples for PK analysis were collected at 0, 0.25, 0.5, 1, 1.5, 2, 3, 4, 6, 10, 24, 48, 72, 96, and 120 h following glasdegib administration, using dipotassium ethylenediaminetetraacetic acid tubes. Glasdegib plasma concentrations were determined at Covance Bioanalytical Services (Shanghai, China) using a validated, sensitive, and specific high-performance liquid chromatography–tandem mass spectrometric (HPLC–MS/MS) method. A 50-µL plasma aliquot was spiked with deuterated internal standard (glasdegib-d_4_), followed by addition of 10% NH_4_OH (aq), extraction with 1000 µL ethyl acetate, and centrifugation. A 400-µL aliquot of the organic layer was evaporated to dryness under a stream of nitrogen, and the residue was reconstituted with 400 µL of 0.1% formic acid in acetonitrile:water (25:75 v/v) and injected into the HPLC–MS/MS system. Chromatographic separation was achieved with a Zorbax XDB-C18 (50 × 2.1 mm, 5 µm; Agilent Technologies, Santa Clara, CA) HPLC column heated to 30 °C and a mobile-phase gradient at a flow rate of 600 µL/min. Mobile phase A consisted of 0.1% formic acid in water and mobile phase B consisted of 0.1% formic acid in acetonitrile. The mobile-phase composition started at 20% B for 0.4 min and increased linearly to 75% B over 1.6 min. Detection of glasdegib and the internal standard was by MS/MS (Sciex API 4000; Applied Biosystems, Foster City, CA) in multiple reaction monitoring mode using positive ion electrospray (IonSpray voltage of 3000 V and temperature at 550 °C). The monitored ion transitions were *m/z* 375 → 257 for glasdegib and *m/z* 379 → 257 for the internal standard.

Calibration curves were linear over the range of 3–3000 ng/mL for glasdegib in plasma, using a weighted (1/concentration^2^) linear regression. The lower limit of quantification (LLOQ) of glasdegib was 3 ng/mL. PK plasma samples were stored at − 70 °C and assayed within the 575 days of established frozen plasma stability. Inter-assay accuracy (percent relative error) at 9, 100, and 2250 ng/mL glasdegib in quality control samples ranged from − 0.4 to 2.0%. Inter-assay precision [percent coefficient of variation (%CV)] was ≤ 6.1% across quality control levels.

Glasdegib PK parameters were calculated using noncompartmental analysis of plasma concentration–time data. Samples below LLOQ were set to 0 for analysis. Actual sample collection times were used for the PK analysis. *C*_max_ and *T*_max_ were the observed values. AUC_last_ was determined using the linear/log trapezoidal method. AUC_inf_ was calculated as AUC_last_ + (*C*_last_/*K*_el_), where *C*_last_ was the predicted plasma concentration at the last quantifiable time point estimated from the log-linear regression analysis and *K*_el_ was the terminal phase rate constant calculated by a linear regression of the log-linear concentration-time curve. The *t*_½_ was calculated as log_e_(2)/*K*_el_, CL/*F* was calculated as dose/AUC_inf_, and *V*_*z*_*/F* was calculated as dose/(AUC_inf_ × *K*_el_).

### Safety assessments

All subjects received glasdegib treatment and were included in the safety analyses. Safety and tolerability of glasdegib were assessed by adverse event (AE) monitoring and changes in clinical laboratory results, ECGs, and physical examination findings. AEs were graded according to the Medical Dictionary for Regulatory Activities version 20.0.

### Sample size determination

For bioequivalence, a sample size of 24 subjects provided (i) 98% power, so that the 90% confidence interval (CI) for the ratio of test-to-reference treatment for glasdegib AUC_inf_ would be within the acceptance range of 80‒125% and (ii) 92% power, so that the 90% CI for the ratio of test-to-reference treatment for *C*_max_ would be within the acceptance range of 80‒125%. This estimate was based on the assumption that the true ratio of test-to-reference for both AUC_inf_ and *C*_max_ was 1.05, within-subject standard deviations of 0.144 and 0.179 for log_e_ AUC_inf_ and log_e_*C*_max_, respectively, obtained from the mean of three prior studies of glasdegib [8, 9, 12].

The sample size of 12 subjects per group for assessment of the effect of food or a PPI on glasdegib PK was chosen empirically, as these assessments were for estimation purposes. A sample size of 12 subjects provided 90% CIs for the difference between treatments of ± 0.1323 and ± 0.1645 on the natural log scale for AUC_inf_ and *C*_max_, respectively, with 90% coverage probability.

### Statistical analysis

To determine the bioequivalence of ICH glasdegib to di-HCl glasdegib, natural log-transformed AUC_inf_, AUC_last_, and *C*_max_ for glasdegib were analyzed using a mixed-effects model with sequence, period, and treatment as fixed effects and subject within sequence as a random effect. To estimate the effects of food and PPIs on the bioavailability of glasdegib, natural log-transformed AUC_inf_, AUC_last_, and *C*_max_ for glasdegib were analyzed using a mixed-effects model with treatment as a fixed effect and subject as a random effect. Adjusted mean differences and 90% CIs for the differences from the models were exponentiated to provide estimates of the adjusted geometric mean ratio and 90% CIs (Test:Reference).

## Results

### Subjects and baseline characteristics

A total of 24 subjects were enrolled in the study, and six subjects were randomized to each of the four sequences. All enrolled subjects received glasdegib treatment and completed the study. The mean age of subjects was 37 years (range 25‒53 years) and the majority of subjects were black (*n* = 17). All but one subject was male. The mean weight was 82.7 kg (range 58.5‒95.7 kg), with a mean height of 175.7 cm (range 165‒189 cm). The mean BMI was 26.8 kg/m^2^ (range 19.3‒30.5 kg/m^2^).

### Pharmacokinetic results

#### Bioequivalence of ICH glasdegib

**Fig. 2:**
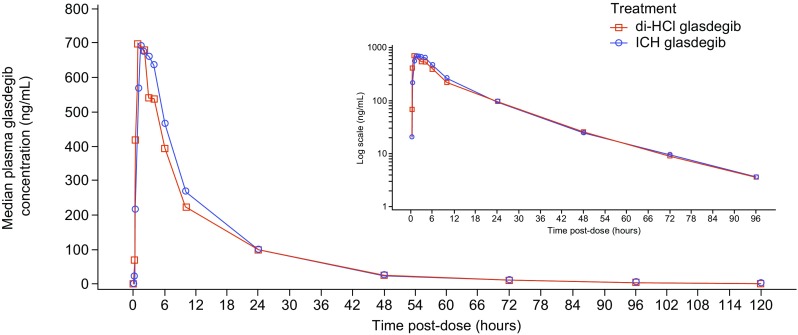
Pharmacokinetics of ICH glasdegib and di-HCl glasdegib. Linear median glasdegib plasma concentration–time profiles for both glasdegib formulations (inset contains same data as semi-log profile)

The PK parameters for ICH glasdegib and di-HCl glasdegib under fasted conditions are summarized in Table 1. The median plasma glasdegib concentration–time profiles for both formulations are presented in Fig. 2a. The ratios (ICH glasdegib:di-HCl glasdegib) of adjusted geometric means of glasdegib AUC_inf_ and *C*_max_ were 104.0% (90% CI 99.7‒108.5%) and 101.6% (90% CI 96.1‒107.4%), respectively (Table 1). The corresponding 90% CIs for the ratios of adjusted geometric means were contained within the acceptance range for bioequivalence (80‒125%). The median (range) *T*_max_ was 1.0 (0.5–3.0) h for di-HCl glasdegib and 2.0 (0.5–4.0) h for ICH glasdegib. The apparent terminal *t*_½_ values were similar for the two treatments, with mean values of 14.99 h and 15.20 h for di-HCl glasdegib and ICH glasdegib, respectively. Inter-subject variability for AUC_inf_ and *C*_max_ for the two formulations was similar, with geometric %CV ranging 30–31% for AUC_inf_ and 28–30% for *C*_max_. No sequence or period effects were observed for *C*_max_, AUC_last_, and AUC_inf_.

**Table 1:** Summary statistics for plasma glasdegib PK parameters by treatment

Treatment	Parameter/comparison	*n*	AUC_inf_ (ng·h/mL)	AUC_last_ (ng·h/mL)	*C*_max_ (ng/mL)	*T*_max_ (h)^a^	CL/*F* (L/h)	*Vz/F* (L)	*t*_½_ (h)^b^
Di-HCl glasdegib 100 mg (fasted)	Geometric mean (geometric %CV)	24	8368 (31)	8275 (31)	752.0 (28)	1.0 (0.5–3.0)	11.95 (31)	253.6 (22)	14.99 ± 3.04
ICH glasdegib 100 mg (fasted)	Geometric mean (geometric %CV)	24	8704 (30)	8612 (30)	764.3 (30)	2.0 (0.5–4.0)	11.48 (30)	246.1 (23)	15.20 ± 3.22
	Adjusted geometric mean ratio (90% CI)^c^		104.0 (99.7‒108.5)	104.1 (99.7‒108.6)	101.6 (96.1‒107.4)				
ICH glasdegib 100 mg (fed)	Geometric mean (geometric %CV)	12	7927 (24)	7833 (24)	560.5 (33)	3.0 (1.0–6.0)	12.62 (24)	289.0 (22)	16.08 ± 2.67
	Adjusted geometric mean ratio (90% CI)^d^		84.3 (78.6‒90.6)	84.1 (78.2‒90.5)	69.0 (61.8‒77.0)				
ICH glasdegib 100 mg + PPI (fasted)	Geometric mean (geometric %CV)	12	8110 (42)	8006 (42)	587.3 (27)	3.0 (1.5–6.0)	12.34 (42)	260.2 (20)	15.39 ± 5.26
	Adjusted geometric mean ratio (90% CI)^e^		100.6 (93.2‒108.6)	100.5 (93.1‒108.5)	80.5 (70.7‒91.6)				

*AUC*_inf_ area under the plasma concentration–time curve from time zero to infinity, *AUC*_last_ area under the plasma concentration–time profile from time 0 to the last quantifiable concentration, *CI* confidence interval, *CL*/*F* apparent oral clearance, *C*_max_ maximum plasma concentration, *CV* coefficient of variance, *t*_½_ terminal half-life, *PK* pharmacokinetic, *PPI* proton-pump inhibitor (rabeprazole), *T*_max_ time to first occurrence of *C*_max_, *V*_*z*_/*F* apparent volume of distribution

^a^Median (range) for *T*_max_

^b^Arithmetic mean (± SD) for *t*_½_

^c^Ratio: ICH glasdegib 100 mg:di-HCl glasdegib 100 mg

^d^Ratio: ICH glasdegib 100 mg (fed):ICH glasdegib 100 mg

^e^Ratio: ICH glasdegib 100 mg + PPI:ICH glasdegib 100 mg

### Effect of food

**Fig. 3:**
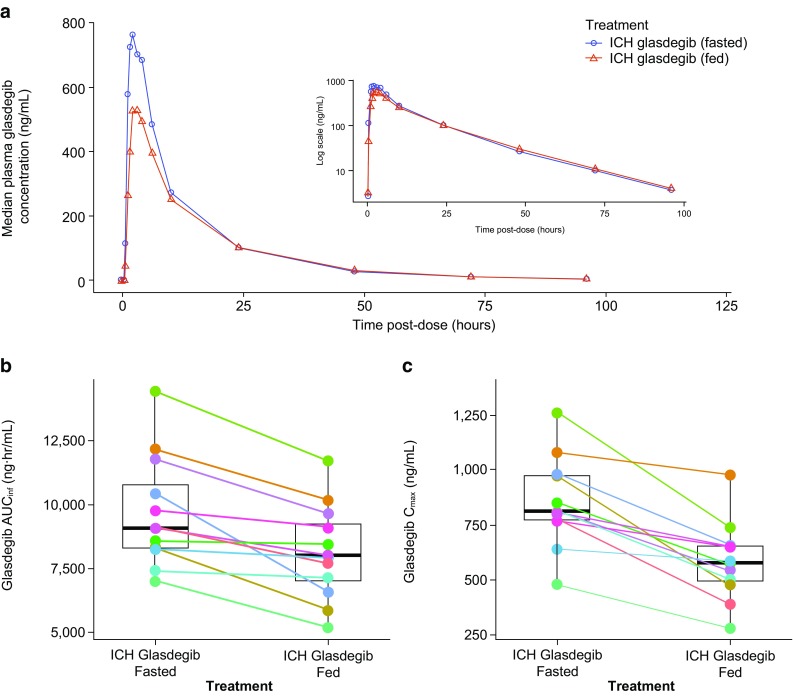
Effect of food on ICH glasdegib. **a** Linear median glasdegib plasma concentration–time profiles for glasdegib given under fasted and fed conditions (inset contains same data as semi-log profile), **b** Matchstick plots for change in exposure for each subject when ICH glasdegib is given under fasted and fed conditions for AUC_inf_, and **c***C*_max_

The median plasma concentration–time profiles for a single dose of ICH glasdegib administered following either overnight fasting (fasted) or after a high-fat, high-calorie meal (fed) are presented in Fig. 3a. Changes in AUC_inf_ and *C*_max_ due to food effect for each subject are provided in Fig. 3b, c. The PK parameters for ICH glasdegib under fed conditions are summarized in Table 1. The ratios [ICH glasdegib (fed):ICH glasdegib (fasted)] of adjusted geometric mean ratio (90% CIs) of AUC_inf_ and *C*_max_ were 84.3% (78.6‒90.6%) and 69.0% (61.8‒77.0%), respectively (Table 1). The observed median *T*_max_ (range) post-dose was 3.0 (1.0–6.0) h under fed conditions and 2.0 (0.5–4.0) h under fasted conditions. The mean apparent terminal t_½_ was similar under both conditions (16.08 vs. 15.20 h, ICH glasdegib fed vs. fasted). Inter-subject variability for glasdegib exposure based on geometric %CV ranged from 24 to 30% for AUC_inf_ and from 30 to 33% for *C*_max_ for the effect of food on treatment.

### Effect of a PPI

**Fig. 4:**
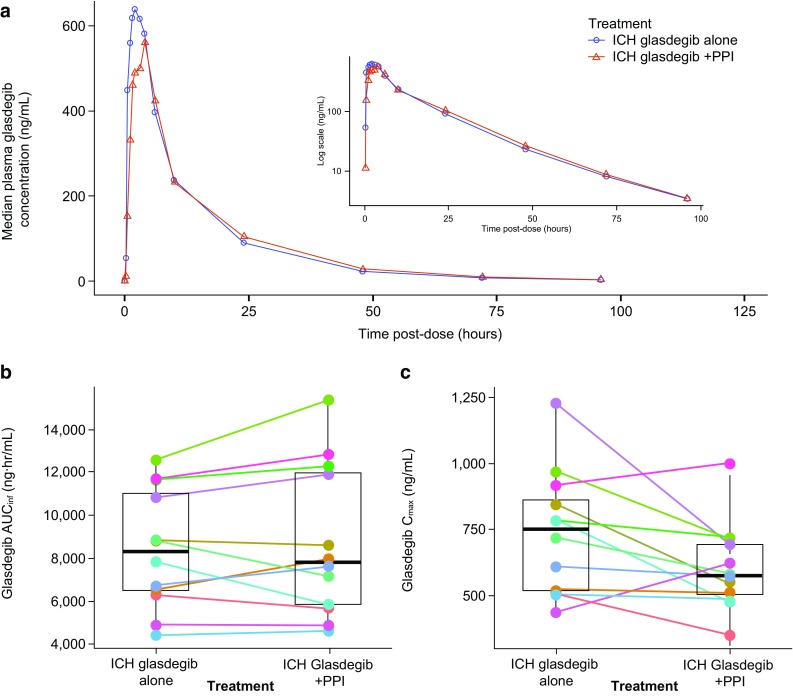
Effect of PPI (rabeprazole) on ICH glasdegib. **a** Linear median glasdegib plasma concentration–time profiles for glasdegib given alone and with a PPI (inset contains same data as semi-log profile), **b** Matchstick plots for change in exposure for each subject when ICH glasdegib is given with and without a PPI for AUC_inf_, and **c***C*_max_

The median plasma glasdegib concentration–time profiles for subjects who received or did not receive a PPI (rabeprazole) are presented in Fig. 4a. Changes in AUC_inf_ and *C*_max_ due to PPI effect for each subject are provided in Fig. 4b, c. PK parameters for ICH glasdegib administered with a PPI are summarized in Table 1. The ratios [ICH glasdegib + PPI:ICH glasdegib (fasted)] of adjusted geometric means (90% CIs) of AUC_inf_ and *C*_max_ were 100.6% (93.2‒108.6%) and 80.5% (70.7‒91.6%), respectively (Table 1). The median *T*_max_ (range) post-dose was 3.0 (1.5–6.0) h with PPI treatment, compared with 2.0 (0.5–4.0) h for ICH glasdegib alone. The mean apparent terminal *t*_½_ was similar under both treatments (15.39 vs. 15.20 h, ICH glasdegib with vs. without PPI). Inter-subject variability for glasdegib exposure based on geometric %CV ranged from 30 to 42% for AUC_inf_ and from 27 to 30% for *C*_max_ for PPI effect on treatment.

### Safety

A single 100-mg dose of glasdegib was generally well tolerated when administered under fasted or fed conditions and with a PPI in healthy subjects. The incidence of treatment-emergent AEs (TEAEs) is summarized in Table 2. Overall, 17 TEAEs were reported in 12 subjects across all treatment arms [di-HCl glasdegib, *n* = 5; ICH glasdegib, *n* = 2; ICH glasdegib (fed), *n* = 3; ICH glasdegib + PPI, *n* = 2]. The most commonly observed TEAEs were gastrointestinal disorders (35% of all TEAEs), particularly vomiting. All observed TEAEs were mild, and none were determined by the investigator to be treatment-related. All TEAEs had resolved by the end of the study. There were no deaths, serious AEs, severe AEs, dose reductions, or permanent/temporary discontinuations due to AEs reported in this study.

**Table 2:** Incidence of treatment-emergent AEs, all causalities

	Di-HCl glasdegib 100 mg (*n* = 24)	ICH glasdegib 100 mg (*n* = 24)	ICH glasdegib 100 mg fed (*n* = 12)	ICH glasdegib 100 mg + PPI (*n* = 12)
Subjects with any AE, *n*	5	2	3	2
Gastrointestinal disorders, *n*	1	1	2	2
Diarrhea	0	1	0	1
Nausea	0	0	1	1
Vomiting	1	0	1	1
General disorders and administration site conditions, *n*	1	1	2	0
Non-cardiac chest pain	1	0	1	0
Vessel puncture site hemorrhage	0	1	1	0
Injury, poisoning and procedural complications, *n*	2	0	0	0
Ligament sprain	1	0	0	0
Procedural pain	1	0	0	0
Nervous system disorders, *n*	1	0	1	1
Headache	1	0	0	1
Migraine	0	0	1	0
Skin and subcutaneous tissue disorders, *n*	0	0	0	1
Skin irritation	0	0	0	1
Total preferred term events, *n*	5	2	5	5

Includes all data collected since the first dose of study drug. Subjects were counted only once per treatment in each row

*AE* adverse event, *PPI* protein pump inhibitor

## Discussion

A new maleate-salt formulation glasdegib tablet that is compliant with the ICH guidelines was developed following an earlier study that determined an active pharmaceutical ingredient particle size for the manufacture of maleate glasdegib tablet formulations [8]. The primary goal of this study was to determine the bioequivalence of the new ICH glasdegib formulation to the di-HCl formulation used in previous Phase I/II trials, including the Phase II clinical trial that generated efficacy and safety data used in glasdegib filings for regulatory approval for treatment of patients with AML or MDS [13]. In these early clinical trials, glasdegib was initially administered in the fasted state, with no food allowed 2 h before and 2 h after the daily dose, and eventually tested irrespective of food consumption [7, 12, 13]. The new tablet formulation is physically more stable than the previously tested di-HCl glasdegib tablet and is intended to be the final commercial formulation. This study determined the 100-mg ICH maleate glasdegib formulation to be bioequivalent to the 100-mg di-HCl tablet formulation under fasted conditions because the 90% CI for the ratios (ICH glasdegib/di-HCl glasdegib) of the adjusted geometric mean ratio fell wholly within the acceptance interval of 80–125% for both AUC_inf_ and *C*_max_.

This study was designed to estimate the maximal effect of food on the PK of oral ICH glasdegib by evaluating the effect of a high-fat, high-calorie meal consumption immediately prior to glasdegib administration [14]. Food ingestion can change the bioavailability of oral medications by various means, including delaying gastric emptying, stimulation of bile flow, changes in gastrointestinal pH, increase of splanchnic blood flow, changes in luminal metabolism of the studied drug, or physical or chemical food interactions with the drug product [14]. Administration of ICH glasdegib in the presence of a high-fat, high-calorie meal resulted in a 16% decrease in geometric mean AUC_inf_ and 31% decrease in geometric mean *C*_max_ compared with administration under fasted conditions (Table 1; Fig. 3a). The change in both AUC_ inf_ and *C*_max_ in individual subjects was relatively consistent (Fig. 3b, c). There was a minimal difference in the median *T*_max_ in the presence of food, wherein the range of *T*_max_ was comparable with and without food. These results are similar to those previously reported for the effect of food on the di-HCl tablet formulation and a previous maleate tablet formulation [8, 9]. Based on preclinical studies, the efficacy of glasdegib is considered to be due to the continuous inhibition of the Hedgehog pathway. Therefore, small changes in overall exposure (AUC_inf_) are likely not clinically significant [9]. Inhibition of the Hedgehog pathway in skin has typically been used to measure the pharmacodynamics of smoothened inhibitors. With glasdegib, consistent downregulation of the Hedgehog pathway has been observed at the 50 mg QD dose, indicating the modulation of pathway could be maintained in the scenario of lowered exposure due to food [15].

Additionally, in the first-in-patient study in patients with hematologic malignancies, signs of clinical activity were noted over a wide range of dose levels tested [5]. The clinical efficacy of the clinical dose of 100 mg QD was further established in a randomized Phase II study in patients with AML or high-risk MDS dosed with glasdegib without regard to food and use of pH altering agents that demonstrated survival benefit when in combination with chemotherapy compared with chemotherapy alone [16]. Therefore, food appeared to have a minimal effect on glasdegib exposure (16% reduction in AUC_inf_) following a single oral dose, and its impact on the PK of glasdegib was not considered clinically meaningful.

The solubility of glasdegib, a weakly basic drug, is pH-dependent, with higher solubility at acidic pH that drops substantially at pH ≥ 6.3. Therefore, PPIs may affect the solubility of co-administered drugs by elevating gastric pH, potentially resulting in reduced bioavailability. Healthy volunteers received a PPI (rabeprazole) over a 7-day period prior to dosing with glasdegib, ensuring an elevated gastric pH at the time of glasdegib administration [17]. Administration of glasdegib in the presence of a PPI did not result in a change in geometric mean AUC_inf_, whereas there was a decrease (~ 20%) in geometric mean *C*_max_ compared with administration under fasted conditions (Table 1; Fig. 4a). The change in both AUC_inf_ and *C*_max_ in individual subjects was inconsistent (Fig. 4b, c), with increases, decreases, and no change observed in different subjects. There was minimal difference in the median *T*_max_ in the presence of PPI compared with the half-life of glasdegib, wherein the range of *T*_max_ was approximately similar with vs. without PPI. These findings are similar to results previously reported for the effect of PPI on the tablet formulation [8]. Overall, treatment with a PPI had no clinically meaningful effect (AUC_inf_, 100.6%) on the plasma PK of 100-mg ICH glasdegib.

A single oral dose of glasdegib was well tolerated, and all AEs observed were mild in fasted or fed states and not considered treatment-related in healthy subjects. The most frequently reported AEs were gastrointestinal disorders (diarrhea, vomiting, and nausea) that were resolved by the end of the study.

In conclusion, 100-mg ICH glasdegib formulation tablet, tested under fasted conditions, is bioequivalent to the clinical 100-mg di-HCl glasdegib formulation used in the study that generated efficacy and safety data used for regulatory submission. The results from this clinical study in healthy volunteers demonstrated that food and agents that increase gastric pH (rabeprazole) had a minimal effect on glasdegib exposure, and therefore not considered to be clinically relevant. Additionally, ICH glasdegib was generally safe and well tolerated under all tested conditions. Therefore, ICH glasdegib may be taken irrespective of food intake and administration of pH-increasing agents, which simplifies dosing recommendations and may facilitate compliance in patients with cancer.
